# Supplementary material for: The causal relationship between the human gut microbiota and pyogenic arthritis: a Mendelian randomization study
Source: Front Cell Infect Microbiol. 2024 Nov 26;14:1452480. doi: 10.3389/fcimb.2024.1452480 (PMC11629706; doi:10.3389/fcimb.2024.1452480)
Supplement: Supplementary file 1 [file DataSheet1.zip › mendelian test/ebi-a-GCST90027789.csv_leave_one_out.pdf]

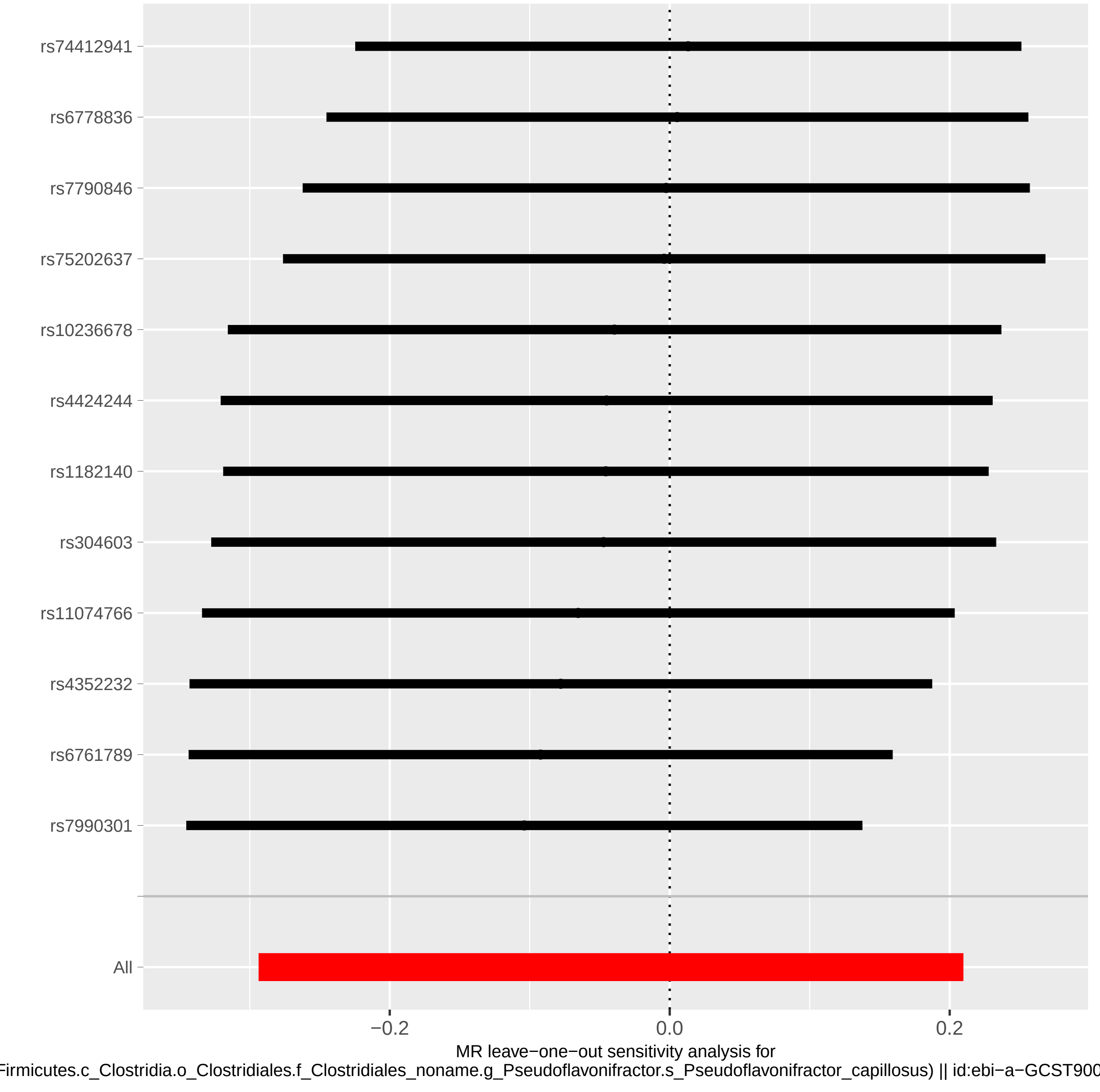
rs74412941
rs6778836
rs7790846
rs75202637
rs10236678
rs4424244
rs1182140
rs304603
rs11074766
rs4352232
rs6761789
rs7990301
All
−0.2
0.0
0.2
MR leave−one−out sensitivity analysis for
Firmicutes.c_Clostridia.o_Clostridiales.f_Clostridiales_noname.g_Pseudoflavonifractor.s_Pseudoflavonifractor_capillosus) || id:ebi−a−GCST900
